# Metabolic reprogramming and immunological changes in the microenvironment of esophageal cancer: future directions and prospects

**DOI:** 10.3389/fimmu.2025.1524801

**Published:** 2025-01-24

**Authors:** Zhi-Xun Guo, Jia-Li Ma, Jin-Qiu Zhang, Ling-Ling Yan, Ying Zhou, Xin-li Mao, Shao-Wei Li, Xian-Bin Zhou

**Affiliations:** ^1^ Taizhou Hospital of Zhejiang Province affiliated to Wenzhou Medical University, Linhai, Zhejiang, China; ^2^ Department of Gastroenterology, Taizhou Hospital of Zhejiang Province affiliated to Wenzhou Medical University, Linhai, Zhejiang, China; ^3^ Key Laboratory of Minimally Invasive Techniques & Rapid Rehabilitation of Digestive System Tumor of Zhejiang Province, Linhai, Zhejiang, China; ^4^ Institute of Digestive Disease, Taizhou Hospital of Zhejiang Province Affiliated to Wenzhou Medical University, Linhai, China

**Keywords:** esophageal cancer, tumor microenvironment, metabolic reprogramming, immune cells, glycolysis, targeted therapy

## Abstract

**Background:**

Esophageal cancer (EC) is the seventh-most prevalent cancer worldwide and is a significant contributor to cancer-related mortality. Metabolic reprogramming in tumors frequently coincides with aberrant immune function alterations, and extensive research has demonstrated that perturbations in energy metabolism within the tumor microenvironment influence the occurrence and progression of esophageal cancer. Current treatment modalities for esophageal cancer primarily include encompass chemotherapy and a limited array of targeted therapies, which are hampered by toxicity and drug resistance issues. Immunotherapy, particularly immune checkpoint inhibitors (ICIs) targeting the PD-1/PD-L1 pathway, has exhibited promising results; however, a substantial proportion of patients remain unresponsive. The optimization of these immunotherapies requires further investigation. Mounting evidence underscores the importance of modulating metabolic traits within the tumor microenvironment (TME) to augment anti-tumor immunotherapy.

**Methods:**

We selected relevant studies on the metabolism of the esophageal cancer tumor microenvironment and immune cells based on our searches of MEDLINE and PubMed, focusing on screening experimental articles and reviews related to glucose metabolism, amino acid metabolism, and lipid metabolism, as well their interactions with tumor cells and immune cells, published within the last five years. We analyzed and discussed these studies, while also expressing our own insights and opinions.

**Results:**

A total of 137 articles were included in the review: 21 articles focused on the tumor microenvironment of esophageal cancer, 33 delved into research related to glucose metabolism and tumor immunology, 30 introduced amino acid metabolism and immune responses, and 17 focused on the relationship between lipid metabolism in the tumor microenvironment and both tumor cells and immune cells.

**Conclusion:**

This article delves into metabolic reprogramming and immune alterations within the TME of EC, systematically synthesizes the metabolic characteristics of the TME, dissects the interactions between tumor and immune cells, and consolidates and harnesses pertinent immunotherapy targets, with the goal of enhancing anti-tumor immunotherapy for esophageal cancer and thereby offering insights into the development of novel therapeutic strategies.

## 1 Introduction

Esophageal cancer (EC) is the seventh-most common cancer and sixth leading cause of cancer-related mortality worldwide. It is a complex multifactorial disease with varying distribution worldwide (1). Among 456,000 annual cases of esophageal cancer, esophageal squamous cell carcinoma (ESCC) comprises approximately 90% of all cases. High-incidence regions include East Asia to Central Asia, the East African Rift Valley, and South Africa, with East Asia having the highest incidence (2). Statistics indicate that China bears over 50% of the global burden of EC cases, with ESCC serving as the primary histological subtype (3).

The emergence, progression, and dissemination of EC are closely related to its cellular microenvironment, also known as the tumor microenvironment (TME). A refractory TME is a key issues that clinically impede effective cancer treatment. Increasing evidence highlights the significance of TME in driving cancer heterogeneity and treatment resistance (4–7). Tumor cells, immune cells, stromal cells, and various cytokines collectively constitute the TME (8), forming a precisely structured ecological environment that favors the expansion, proliferation and dissemination of cancer cells. Immunocompetent cells and supporting stromal elements acting as essential components of the TME, exhibit high specialization and heterogeneity in the phenotype and function, participating throughout the entire process of tumor development and treatment response (9, 10). Metabolic reprogramming plays a crucial role in cancer progression, and the TME is a significant factor influencing this process (11) (**Table 1**).

**Table 1:** Details, key insights, and shortcomings concerning metabolic reprogramming of esophageal cancer and immune cells.

		Key point	Mechanism	Involved factor	Challenge	Article
TME	HIF-1α↓ TAM	Hypoxia-inducible factor (HIF-1α stimulates glycolytic metabolism predominantly in M1-type macrophages, thereby facilitating the inflammatory response.	HIF-1α enhances the NF-κB and PI3K/AKT/mTOR pathways to meet inflammatory energy demands. The expression of glycolytic enzymes (e.g., lactate dehydrogenase, pyruvate dehydrogenase kinase) ↑.	Oxygen concentration, prolyl hydroxylase (PHD) activity, reactive oxygen species (ROS) levels, the NF-κB and AKT/mTOR signaling pathways.; glycolytic enzymes, angiogenic factors like vascular endothelial growth factor receptor (VEGF), and erythropoietin (EPO).	Excessive HIF-1α activation can cause uncontrolled inflammation and tissue injury, linked to macrophage polarization and metabolic dysregulation, making it a promising therapeutic target.	(12)
	Metabolites ↓ TAM	Metabolites produced by tumors, such as lactic acid, adenosine, and prostaglandins promote the polarization of tumor-associated macrophages (TAMs) towards the M2 phenotype via paracrine or autocrine mechanisms.	Under hypoxic conditions, HIF1α is stabilized and activated, resulting in M2-type TAM marker genes (e.g., ARG-1, VEGF) ↑	VEGF facilitates angiogenesis; Arginase-1 (ARG-1) engages in polyamine synthesis and nitrogen metabolism; Lactate transporters (MCTs) facilitate the influx of lactate into TAMs,	Tumor metabolism represents one of numerous signals that govern TAM polarization, highlighting the necessity for personalized treatment strategies to tackle the intrinsic heterogeneity of tumors.	(13)
	Arginine ↓ TME	Arginine plays a crucial role in TME metabolic reprogramming by serving as a precursor for the synthesis of proteins, nitric oxide, polyamines, agmatine, creatine, and urea.	tumor cells are unable to synthesize arginine due to argininosuccinate synthetase 1 (ASS1) deficiency, and instead upregulate insulin-like growth factor 1 (IGF-1R) and enhance ASS1 transcription via c-MYC for arginine metabolic reprogramming.	ARG-1 degrades arginine, limit its availability and suppress T cell function; inducible nitric oxide synthase (INOS) cooperates with ARG-1 to produce nitric oxide and citrulline, modulating the immune microenvironment.	Supplementing arginine or inhibiting the activity of ARG-1 and INOS could represent promising strategies to augment anti-tumor immune responses but their clinical implementation remains a significant challenge.	(14)
	Glutamine ↓ TME	Glutaminolysis plays a pivotal role as both an energy source and nitrogen supplier for the activation and sustained functionality of immune cells.	Glutamine is first converted into glutamate by glutaminase (GLS), then enters the tricarboxylic acid cycle (TCA) to generate energy or is transformed into various biosynthetic precursors for proliferation, differentiation, and cytokine production of immune cells.	Glutaminase (GLS), Glutamine transporters (such as SLC1A5); Activation of the mTOR signaling pathway, Expression of immune checkpoint molecules (such as PD-L1).	Balancing competitive glutamine consumption in the TME to inhibit tumor growth while mitigating impacts on immune cells poses a significant research challenge.	(15)
Immune Cells	Metabolites ↓ T Cell	Lactate is not only an energy source generated through glycolysis, but also plays a crucial role in T cell function by acting as a signaling molecule and regulating signaling pathways.	glucose transporters↑ and glycolytic enzymes↑ fuels T-cell proliferation and activation.; MCTs transport lactate into T cells, altering the NAD+/NADH ratio, regulating silent information regulator 1 (SIRT1) activity, impacting T-bet stability, and modulating gene expression and signaling via lactylation.	Hexokinase 2 (HK2), lactate dehydrogenase A (LDHA), etc.; glucose transporter 1 (GLUT1); monocarboxylate transporter 1 (MCT1), etc.; HIF-1α, Myc, T-bet, SIRT1, etc.; adenylate kinase 2 (AK2), pyruvate kinase isozyme typeM2 (PKM2), etc.	Lactate has dual effects: immunosuppressive at low concentrations.; potentially pro-antitumor for CD8+ T cells at high concentrations. Lactylation still warrant further research into its regulatory mechanisms.	(16)
	lipid metabolism ↓ T cell	Distinct lipid molecules in lipid metabolism are crucial for T cell signaling and function, serving as energy sources, membrane components, and signaling molecules.	Lipid metabolism generates fatty acids, cholesterol, and phospholipids, which modulate transcription factor activity, epigenetic modifications, and post-translational protein modifications, ultimately impacting T cell function and differentiation.	Fatty acid synthase (FASN), acetyl-CoA carboxylase (ACC), fand sterol regulatory element-binding proteins (SREBPs), etc.; phosphatidylinositol, diacylglycerol, sphingomyelin, and cholesterol, etc.	Challenges persist in identifying specific lipid biomarkers for effective monitoring and prediction of immune cell function and treatment response.	(17)

↑ means an increase in the expression; ↓ means the impact of metabolism on TME and immune cells.

Tumors activate immune and stromal cells through immune metabolic reprogramming, such as tumor-associated macrophages (TAMs) (18), tumor-associated neutrophils (TANs) (19), regulatory T cells (Tregs) (20), myeloid-derived suppressor cells (MDSCs) (21), endothelial cells (ECs) (22), and cancer-associated fibroblasts (CAFs) (23). Tumors promote their occurrence and development by increasing aerobic glycolysis (24), affecting protein palmitoylation of oncogenes and tumor suppressor genes (25), and altering microenvironmental cellular metabolism such as abnormal fatty acid synthesis and oxidation, as well as competing for oxygen and glucose (26), and essential or non-essential amino acids (27, 28). Investigating the impact of TME metabolism on stromal and immune cells can significantly enhance our understanding of EC progression and facilitate the development of more effective EC treatment methods.

Chemotherapy remains the primary treatment method for most patients with EC, although it is associated with dose-limiting toxicity (29). Significant advancements in targeted therapy have led to better treatment strategies. However, the National Comprehensive Cancer Network (NCCN) advocates only for the administration of trastuzumab, which specifically targets human epidermal growth factor receptor 2 (HER2), and ramucirumab, an inhibitor of vascular endothelial growth factor receptor 2 (VEGFR-2), as therapeutic options for patients diagnosed with EC (30). In recent years, findings concerning the efficacy of immunotherapy for the treatment of diverse types of cancer have been exciting. For instance, immune checkpoint inhibitors (ICIs), immune-modulating compounds, monospecific antibodies and other immunotherapies represent new methods for treating EC (31, 32). This approach harnesses the patient’s own immune system to fight malignant cells by blocking the immune checkpoint pathways. Inhibiting programmed death 1 (PD-1) and programmed death ligand 1 (PD-L1), classic ICIs, have demonstrated compelling clinical benefits in various malignancies, including ESCC (33, 34). However, despite the availability of numerous treatment options for EC, there is a dearth of effective treatments.

Given the above, we attempted to augment anti-tumor immunotherapy for EC through a comprehensive summation of the metabolic characteristics within the TME, and identify and utilize relevant immunotherapy targets.

## 2 Microenvironment of EC

Within the TME, anti-tumor cells and tumor cells counteract each other. Various molecules released by stromal cells facilitate tumor growth by directly activating cancer cell growth signals or by remodeling the microenvironment (35). Upon activation, immune cells, such as dendritic cells (DCs) (36), effector T cells (T effs) (37), memory T cells (T mems) (38), and natural killer cells (NKs) (39), mount an immune response against tumor cells in the TME, thereby controlling tumor progression and preventing evasion of immune surveillance.

In EC, vascular endothelial growth factor receptor (VEGF) plays a crucial role in tumor progression. Shimada et al. identified a positive correlation between serum VEGF levels and the tumor stage and prognosis by comparing the serum VEGF content in patients diagnosed with ESCC (40). Cancer-associated fibroblasts (CAFs) also constitute a vital component of the EC TME, playing significant roles in the disease progression and prognosis. During disease progression, inhibition of the transcription factor KLF4 in epithelial cells results in a marked reduction in the expression of ANXA1, which serves as a ligand for formyl peptide receptor type 2 (FPR2). This decrease subsequently triggers the unregulated conversion of normal fibroblasts into CAFs, ultimately facilitating crosstalk (41). Cancer-derived S100A8 engages with CD147 receptors on CAFs, triggering their polarization and subsequently fostering chemoresistance via the activation of the intracellular RhoA-ROCK-MLC2-MRTF-A signaling pathway (42). Chen et al. discovered that precancerous esophageal epithelial cells could reprogram normal resident fibroblasts into CAFs by downregulating the ANXA1-FRP2 signaling pathway (43). Qiu et al. emphasized the future prospects and clinical trends of targeting CAFs for the ESCC treatment. A deeper understanding of the molecular biology of CAFs could potentially contribute to the development of novel anti-ESCC strategies (44). Concurrently, prolonged exposure of the esophagus to a gastric acid environment fosters a chronic inflammatory microenvironment. Evidence supports the crucial role of CAFs in the progression of Barrett’s esophagus (BE) and esophageal adenocarcinoma (EAC) (45, 46). In EAC, the distal esophageal epithelium undergoes metaplasia as a consequence of reflux disease, and inflammatory cytokines are significantly upregulated, affecting the cancer prognosis. The infiltration of M2-macrophages, especially the predominance of M2-like cells over M1-like cells, correlates with an unfavorable prognosis (47, 48). In EAC and ESCC, tumor-infiltrating macrophages appear to play a crucial role in the aggressive progression of malignancy and resistance to therapy (49). Some studies have indicated that MDSCs play a role in fostering the growth of esophageal tumors in experimental settings, and elevated infiltration levels of these cells in patients with EC correlate with adverse prognostic indicators (50, 51). This suggests that MDSCs may serve as a potential oncogenic factor within the TME of EC.

Some bacteria also influence the TME in EC. Certain bacterial infections in the TME activate immune responses that directly eliminate tumor cells, while other bacteria induce immune evasion of tumor cells by inhibiting inflammatory pathways (52, 53). Examples of bacteria that influence the TME in EC include Helicobacter pylori (54) and Streptococcus (55).

**Figure 1:**
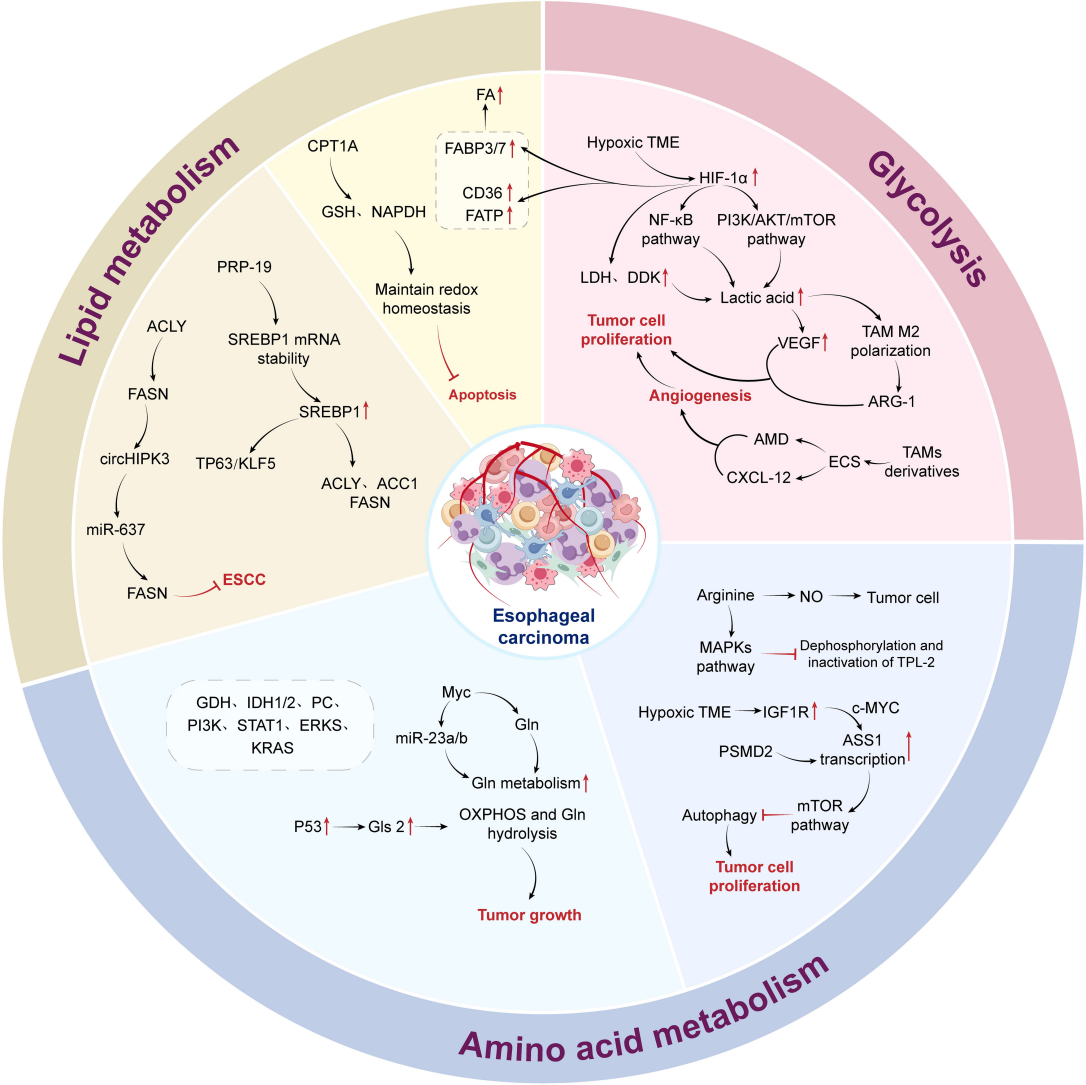
Metabolic reprogramming in the tumor microenvironment of esophageal cancer.

The interaction between neoplastic cells and tumor-suppressing immune cells in the local microenvironment of esophageal tumors reveals meaningful therapeutic strategies. By selectively upregulating or downregulating anti-tumor substances, specifically inhibiting tumor cells, and activating or restoring immune cell functions, an anti-TME is created. Therefore, we have summarized the characteristics of metabolic reprogramming in the esophageal cancer EC TME, providing strategies for the treatment of EC (**Figure 1**).

In this section, we have summarized 21 articles, while 10 discussed immune cells in the TME of EC, 7 explored the roles of VEGF and CAFs in the progression of EC, and 4 investigated the impact of microflora on the esophageal TME.

## 3 Glucose metabolism

In 1956, Otto Warburg noted that, unlike normal cells, cells predominantly utilize glycolysis for energy production, even under aerobic conditions, rather than oxidative phosphorylation (OXPHOS). This metabolic phenomenon is referred to as aerobic glycolysis, also known as the “Warburg effect (56, 57). The increase in aerobic glycolysis results in glucose deprivation and lactate accumulation of lactate within tumor cells. Meanwhile, numerous dysfunctional tumor blood vessels and rapidly proliferating tumor cells create a hypoxic TME, forcing cells to upregulate the expression of glucose transporter 1 (GLUT-1) to counteract glucose consumption by tumor cells and further resulting in an acidic and hypoxic TME (58–60). High glycolytic activity and poor blood exchange reduce glucose availability (61), exacerbating a vicious cycle. This indicates that the improvement of hypoxia and excessive glucose levels can be used as a target to inhibit tumor progression.

Hypoxia-inducible factor (HIF-1α) is expressed in the hypoxic TME and acts as a transcription factor involved in the transcription of glycolytic or glucose transporter genes (59, 62, 63). The transcription of HIF-1α significantly enhances the two major pathways of NF-κB and PI3K/AKT/mTOR, allowing cells to increase glucose metabolism under oxygen-independent conditions (12). Pyruvate kinase isozyme typeM2 (PKM2) is highly expressed in both tumor cells and tumor-associated fibroblasts (64) and is upregulated in patients as well (65). Some studies have revealed that the interplay between PKM2, heat shock protein 90 (HSP90), and HIF-1α leads to the stabilization of PKM2, which in turn facilitates aerobic glycolysis and inhibits the process of cell apoptosis (66). The observed correlation between an elevated PKM2 expression and an adverse prognosis further supports the crucial role of glycolysis in the progression of ESCC.

Lactate dehydrogenase (LDH), pyruvate dehydrogenase (PDH), and pyruvate dehydrogenase kinase (PDK) are key enzymes in pyruvate metabolism. HIF-1α upregulates the expression of LDH and PDK, catalyzing the conversion of pyruvate to lactate and leading to further lactate accumulation (12, 59, 67, 68). Lactate further mediates the expression of VEGF and M2 polarization of TAMs through HIF-1α (69), creating immunosuppressive interactions within TAMs. The accumulation of lactate caused by a hypoxic microenvironment is a crucial factor in reducing anti-tumor immunity.

In some case of tumor progression, the TME can promote the transformation of macrophages into TAMs (70). In a TME characterized by low glucose levels, TAMs modulate their functions through glycolysis activation, while lactate promotes the polarization of M2-type TAMs, leading to elevated expression of VEGF and arginase-1 (ARG-1) and thereby stimulating cancer cell proliferation (71). TAMs indirectly increase the bioavailability of targeted nutrients in the TME, providing nutrition for malignant cells, and their immunosuppressive effects also promote tumor progression (72). The nutritional support mechanism involves the recruitment of TAM-secreted bioactive molecules or activation of the ECs, leading to the generation of TAM-derived adrenomedullin (AMD), chemokine (C-X-C motif) ligand 12 (CXCL12), and other factors, which promote new blood vessel formation (73, 74). Therefore, the role of TAMs in oncogenesis promotion and immunodepression makes them potential targets for cancer therapies.

Glucose is a crucial energy source for activating T-cells, and a low-glucose TME environment can inhibit the T-cell function and reduce their persistence during the initiation of adaptive immune responses (75). As glycolysis increases in tumor cells and TAMs, the abundance of glucose in the TME decreases, eliciting impairment of the T-cell function and further exacerbating the immunosuppressive capacity of the TME. Lactate produced by cancer cells stabilizes extracellular signal-regulated kinases 1/2 (ERK1/2), signal transducer and activator of transcription (STAT) 3, and HIF-1α to induce ARG-1, thereby inhibiting the T-cell function and promoting tumor growth (69, 76). In addition, enhanced glycolysis in tumor cells increases the secretion of colony-stimulating factor (CSF) and macrophage colony-stimulating factor (M-CSF), further inhibiting the function of T cells (77). At this point, low glucose induces increased expression of FoxP3, promoting the transition from T eff cells to T reg cells (78), while also reprogramming T-lymphocyte metabolic processes to adapt to the glucose-deprived environment, characterized by the suppression of glycolysis and the augmentation of OXPHOS (58).

EC cells secrete transforming growth factor-beta (TGF-β), thereby enhancing the immune tolerance of tumors. Clinical studies have established a significant correlation between TGF-β expression levels and the prognosis of patients with EC (79). The TGF-β signaling pathway is abnormally high in patients (80). TGF-β activates regulatory T cells (Tregs) directly, while simultaneously inhibiting the cytotoxicity of T cells and natural killer cells (NKs), impairing DCs antigen-presenting function, and blocking the differentiation of naïve T cells towards effector T cells (81). A study found that TGF-β is highly expressed in patients treated with conventional chemotherapy regimens, suggesting that chemotherapy may upregulate TGF-β levels, leading to the development of immune resistance (80). Furthermore, IL-6 upregulation is evident in both ESCC and EAC (82), originating from the TME and expressed or implicated in related pathways across diverse EC phenotypes (83). This upregulation promotes epithelial-to-mesenchymal transition (EMT), clonogenicity, and chemoresistance in EC (83).

In this section, we have reviewed 33 articles on glucose metabolism in the TME of EC, 11 of which were related to the mechanisms of glucose metabolism, 11 discussed the roles of factors such as HIF-1α and TGF-β in EC, and 13 explored the impact of changes in TME glucose metabolism on immune cells.

## 4 Amino acid metabolism

Amino acids, as essential resources and metabolites for the sustenance of cellular life, participate in the TME, and their increased demand supports the rapid proliferation of cancer cells (84). Among amino acids, arginine is a precursor for the synthesis of proteins, polyamines, nitric oxide, creatine, agmatine, and urea (85), and is also a source of NO, an important substance in tumor regulation (86). Nutritional arginine in the TME activates the MAPK pathway by inhibiting dephosphorylation and subsequent inactivation of TPL-2, a tumor-promoting locus of MAPK kinase (87). This finding highlights the important role of arginine metabolism in cancer progression. In the hypoxic TME of ESCC, the receptor tyrosine kinase insulin-like growth factor 1 (IGF-1R) is upregulated and increases the transcription of argininosuccinate synthetase 1 (ASS1) regulated by c-MYC, achieving reprogramming of arginine metabolism (14). In the rate-controlling steps of arginine metabolism, the differences in the levels of ASS1 and argininosuccinate lyase (ASL) between ESCC tissues and their metastatic derivatives suggest that blocking ASS1 or ASL may hinder the proliferation of ESCC at the original site and potentiate distant metastatic dissemination (88).

Glutamine is the predominantly consumed amino acid in tumors, and glutamine addiction is a typical characteristic of cancer (89). Research has shown that tumor cells have a stronger dependence on glutamine (90), which is widely present in EC cells, as well as other tumor cells. Glutamine serves as a precursor for the biosynthesis of arginine or other non-essential amino acids, purines, pyridines, and glucose, and provides nutrition for rapidly proliferating malignant cells. Its redox reaction removes reactive oxygen species, making it indispensable for the survival of cancer cell. Indeed cancer cells that lack glutamine undergo rapid cell death, making glutamine a crucial target for cancer therapy (91–93).

The metabolic reprogramming of glutamine is orchestrated by various oncogenic genes (94). Specifically, Myc can directly bind to the promoters of glutamine metabolism genes to enhance glutamine metabolism (95), or indirectly stimulate it by inhibiting the expression of miR-23a/b (96). In contrast, p53 promotes OXPHOS and glutamine hydrolysis, thereby inhibiting tumor growth by upregulating the expression of glutaminase isoenzyme Gls2 (97). Other genes, such as glutamate dehydrogenase (GDH) (98), IDH1/2 (99), pyruvate carboxylase (PC) (98), phosphatidylinositol 3-kinase (PI3K) (100), signal transducer and activator of transcription 1 (STAT1) (101, 102), extracellular signal-regulated kinases (ERKs) (103), and KRAS (104), are associated with metabolic reprogramming of glutamine. In experiments with porcine intestinal ECs, the mTOR and MAPK/ERK signaling pathways were inactivated in a low-glutamine environment and reactivated after glutamine supplementation (105). Glutamine is also essential for T-cell function. Indeed, recent studies have shown that glutamine is a key *in-vivo* fuel for CD8 T-cells (106). Glutamine depletion inhibits T-cell proliferation and cytokines production, and this inhibitory effect on T-cells is irreversible (107).

The competition for glutamine between cancer cells and T cells results in a relative deficiency of glutamine in T cells (108, 109). Therefore, we can combat tumor proliferation by specifically modulating glutamine levels in the TME of EC. For example, the glutaminase (GLS) inhibitor CB839 not only delay tumor proliferation (110), but also enhances the therapeutic effect of chimeric antigen receptor T-cells (CAR-T) (111). The glutamine transporter inhibitor V-9302 has been demonstrated in animal experiments to reduce the uptake of glutamine by malignant cells in mice (112). Edwards et al. further showed that V-9302 not only diminishes the uptake by malignant cells but also augments the infiltration of CD8+ T cells, elevates the count of Th1 cells secreting the anti-tumor effector molecule IFNγ within the TME, and mitigates tumor-induced glutamine deprivation in lymphocytes, ultimately attenuating the impairment of anti-tumor immunity (109). Therefore, we anticipate that new targeted glutaminase drugs will contribute to the management of EC.

In summary, the amino acids in the TME are indispensable for cell metabolism. The proliferative activity and expansion of cancer cells, along with the activation and differentiation of immune cells, demand a high-energy microenvironment. Amino acids, which serve as secondary major energy providers, exert a profound influence on cellular metabolism. In this section, 30 articles were included, while 6 focused on arginine metabolic reprogramming, 19 focused on glutamine metabolic reprogramming, and 5 discussed therapeutic strategies based on amino acid metabolic reprogramming. A deeper understanding of amino acid metabolism in the TME can aid in identifying novel anti-tumor targets and offer new perspectives for targeted therapies.

## 5 Lipid metabolism

Alterations in lipid metabolism represent a significant aspect of cellular reprogramming in cancer and serve as a crucial mode of interaction between tumor cells and the TME. Their synthetic and catabolic processes can be effective targets for lipid metabolism.

ESCC progression requires the enhanced synthesis and uptake of lipids (113). Lipid synthesis metabolism is associated with several enzymes, including ATP citrate lyase (ACLY), acetyl-CoA carboxylase (ACC), fatty acid synthase (FASN), and sterol regulatory element binding protein 1 (SREBP1). ATP citrate lyase (ACLY) catalyzes a key rate-limiting step in the biosynthesis of fatty acids, cholesterol, and other lipids during *de novo* fat synthesis. Research has demonstrated that overexpression of ACLY stimulates the proliferation of tumor cells, whereas suppression of ACLY expression hinders their growth (114–116). FASN serves as the catalyst for the final step of fatty acid production, forming palmitate, which involves the conversion of intermediates into saturated fatty acids in the presence of NADPH. FASN exhibits low expression in quiescent normal cells (117), but is overexpressed in many types of cancer. Elevated expression and activity of FASN contributes to the survival of cancer cells (118) and has implicated in the progression of ESCC. For example, the competitive endogenous RNA circHIPK3 upregulates FASN expression in ESCC cells by sponging miR-637, increasing fatty acid biosynthesis, and promoting tumor progression (118). In hypoxic tumor cells, the upregulation of the hypoxia-inducible factor HIF-1α leads to a subsequent increase in FABP3/7 expression (119). Concurrently, there is a significant elevation in the gene and protein levels of CD36 and FATP in these cells (26). This coordinated upregulation facilitates the influx and accumulation of fatty acids (FAs), ultimately improving the survival of hypoxic tumor cells. Preclinical investigations have revealed that the internalization of exogenous fatty acids by CD36 is contingent on CD36 expression, and notably, the combined application of CD36 inhibitors with FASN inhibitors and anti-PD-1 therapy exhibits marked synergistic efficacy (120).

SREBP1 is an important transcriptional regulator of lipid synthesis that regulates the fat generation process by activating ACLY, ACC1, and FASN (121). It can also synergize with TP63/kruppel-like factor 5 (KLF5) in the regulation of fatty acid biosynthesis (122). SREBP1 overexpression correlates with an unfavorable prognosis in ESCC patients and facilitates ESCC progression through the stimulation of fatty acid biosynthesis. In ESCC, pre-mRNA processing factor 19 (PRP19) enhances the stability of SREBP1 mRNA in an n6-methyladenosine-dependent manner, mediating SREBP-dependent fatty acid synthesis and ESCC progression (122). Therefore, we may be able to reduce fatty acid synthesis in TME and disrupt the energy intake of ESCC by targeting these key enzymes, ultimately achieving the goal of delaying tumor progression.

Fatty acid oxidation (FAO) is a process in which fatty acids are shortened to produce acetyl-CoA, NADH, and FADH2 (123). Carnitine palmitoyl transferase I (CPT1A) is a key rate-limiting enzyme in FAO, facilitating the transport of long-chain fatty acids into the mitochondria for oxidation. Its upregulation in ESCC is associated with low survival rates in patients (124). The concentrations of medium- and long-chain acylcarnitines, which serve as the primary substrates for energy generation through FAO in the mitochondria, are markedly reduced in the peripheral blood of patients with ESCC compared to healthy controls. This reduction suggests alterations in β-oxidation activity and the tricarboxylic acid (TCA) cycle in the ESCC cells (125). In ESCC, CPT1A maintains redox homeostasis by providing GSH and NADPH, thereby inhibiting apoptosis. Inhibiting CPT1A, which leads to a decrease in NADPH supply, can thus inhibit anchorage-independent growth of ESCC cells both *in vitro* and *in vivo* (124). Overexpression of CPT1A activates FAO and is closely correlated with grading, metastasis, clinical staging, and a poor prognosis in EC patients.

At the same time, FAO is also one of the sources of OXPHOS for some immune cells. For example, compared to T eff cells, the sustained upregulation of CPT1A expression in T cells and studies using CPT1A inhibitors indicate that FAO is an important metabolic pathway in T cells (126). The differentiation and activation of TAM are related to FAO (127). By inhibiting mitochondrial OXPHOS, particularly FAO, the tendency for M2 polarization of TAMs is diminished, resulting in reduced tumor proliferation, angiogenesis, and immunosuppression. These observations underscore the potential of CPT1A as a promising target for clinical intervention in ESCC treatment strategies.

**Figure 2:**
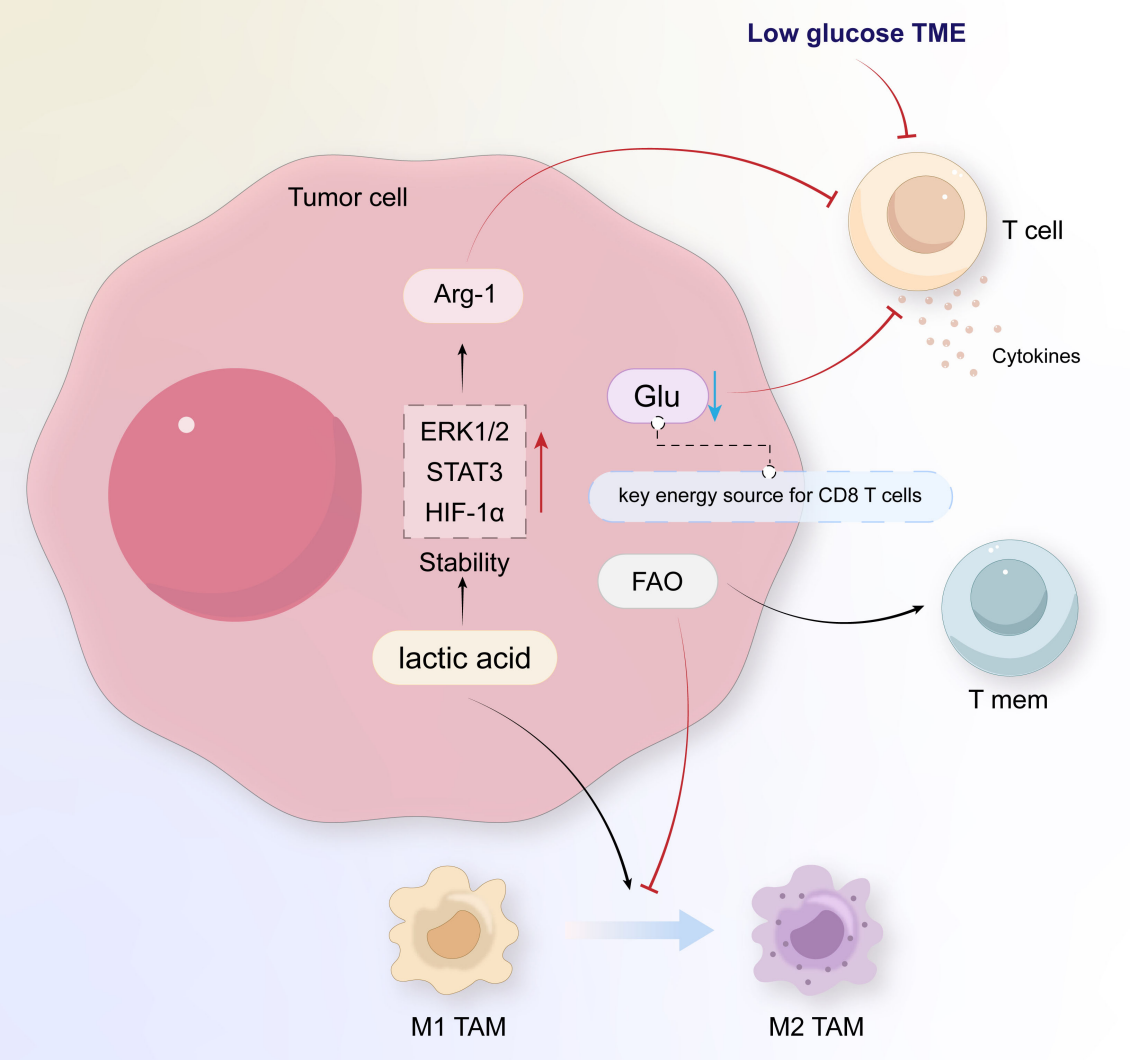
Immunological changes in the tumor microenvironment of esophageal cancer.

The high energy demands of malignant cells necessitate increased intake, resynthesis, and oxidative breakdown of exogenous lipids through additional fat metabolism to obtain energy. This process of energy alteration also exerts an influence on immune cells. In the TME, lipid metabolism synergizes with glucose and amino acid metabolism to alter the normal functions of immune cells, leading to immunosuppression (**Figure 2**). This provides tumors with an opportunity to evade immune defense mechanisms, thereby promoting the development of escape mechanisms and cancer progression. We have selected 17 articles that reference research on the relationship between lipid metabolism in the tumor microenvironment and the interaction between tumor cells and immune cells, 12 of which focused on the anabolic metabolism of lipids, and 5 focused on the oxidative catabolism of lipids, hoping to better integrate the relevant targets of lipid metabolism and provide new directions for the development of new drugs.

## 6 Conclusion and outlook

EC is a complex and malignant disease that involves tumor cells, creating a nutritious environment conducive to tumor growth by activating various pathways. Tumor metabolites and metabolic regulation affect the function of immune cells, thereby resulting in local immunosuppression that enables tumors to evade the host’s immune surveillance mechanisms. Therefore, addressing metabolic abnormalities in the TME is crucial for the initiation and progression of esophageal cancer.

Currently, effective treatment options for EC are limited, and the treatment response and overall survival rates of patients undergoing tumor immunotherapy remain suboptimal. The essential roles of glucose, glutamine, and other nutrients in cell proliferation and immune defense make it challenging to avoid the toxic and side effects associated with targeted modulation of their levels within the TME. However, targeted therapies continue to face the challenge of drug resistance. For instance, an elevated GLS expression in tumor cells enables glutamine synthesis from glutamate, thereby permitting malignant cells to sustain proliferation even during glutamine deprivation (128).

Metabolic reprogramming is a distinctive feature of both tumor cells and immune responses. The immunosuppressive microenvironment in EC tumors, marked by elevated lactate levels, reduced amino acid availability, and increased fatty acid accumulation along with heightened energy consumption, represents a key focus for metabolic interventions in targeted therapies. Several microenvironment-targeted drugs, including PD-1/PD-L1 inhibitors and anti-angiogenic agents, have progressed to clinical trials and shown promising efficacy. Nevertheless, the therapeutic mechanisms underlying metabolic reprogramming in the EC microenvironment are in the preclinical stage. Further exploration of its immunological and biological facets is pivotal for the development of novel drugs and the refinement of existing immunotherapies (**Table 2**).

**Table 2:** Prospects and challenges of EC therapies.

Treatment	Prospect	Challenge
Traditional chemotherapy	Chemotherapy continues to be a cornerstone in the treatment of esophageal cancer, particularly as an adjuvant therapy for patients who are ineligible for surgery or post-surgical care.	Toxic side effects and drug resistance remain difficult to address.
Immunotherapy	Immune checkpoint inhibitors (ICIs), especially PD-1/PD-L1 inhibitors, show promise in treating esophageal cancer with notable anti-tumor activity and safety. Cell therapies like Chimeric Antigen Receptor T-Cell Immunotherapy (CAR-T) enhance T-cell precision in cancer cell recognition and elimination.	Patient responses to immunotherapy demonstrate considerable heterogeneity, and resistance may arise from gene mutations or bypass signaling. Current treatment plans for EC rely mainly on clinicopathological characteristics, with limited consideration of molecular features.
Targeted Therapy	Targeting specific markers in the TME enhances nutrient availability and immune regulation, improving treatment specificity and reducing side effects.	Compared to other cancer types, there are relatively few targeted therapeutic drugs approved for the treatment of esophageal cancer (e.g., human epidermal growth factor receptor 2 (HER2), vascular endothelial growth factor receptor 2 (VEGFR-2)), which necessitates more extensive clinical data for support.

Consequently, we anticipate utilizing more advanced sequencing techniques to delve deeper into the interactions between immune cells and tumor cells in EC metabolism. For instance, the application of spatial tri-omic sequencing technologies enables the delineation of spatial dynamic remodeling within EC (129). Perturb-DBiT has the capacity to elucidate clonal dynamics and cooperative interactions, while also identifying differential and synergistic perturbations that promote or inhibit immune infiltration in tumors (130). In addition, spatial CITE sequencing can reveal spatially distinct germinal center reactions in EC (131). Our goal was to identify suitable biomarkers that can reveal more characteristic targets, ultimately leading to the discovery of more effective novel therapies that can enhance nutrient availability and improve immune regulation within the TME. We should also consider the differences in sensitivity to targeted therapy between EAC and ESCC, as well as the potential risks associated with novel targeted therapies. Furthermore, it is important to consider potential risks associated with novel targeted therapies. By collecting and analyzing effective clinical data, we can explore more effective combinations of different targeted therapies, immunotherapy in conjunction with targeted therapy, and targeted therapy combined with conventional chemotherapy (132). These efforts will aid in the development of new anti-EC drugs and enhance the overall efficacy of EC treatments.

## References

[1] Sung H Ferlay J Siegel RL Laversanne M Soerjomataram I Jemal A. Global cancer statistics 2020: GLOBOCAN estimates of incidence and mortality worldwide for 36 cancers in 185 countries. CA Cancer J Clin. (2021) 71:209–49. doi: 10.3322/caac.21660 33538338

[2] Abnet CC Arnold M Wei W-Q. Epidemiology of esophageal squamous cell carcinoma. Gastroenterology. (2018) 154:360–73. doi: 10.1053/j.gastro.2017.08.023 PMC5836473 28823862

[3] Zhou M Wang H Zeng X Yin P Zhu J Chen W. Mortality, morbidity, and risk factors in China and its provinces, 1990-2017: a systematic analysis for the Global Burden of Disease Study 2017. Lancet Lond Engl. (2019) 394:1145–58. doi: 10.1016/S0140-6736(19)30427-1 PMC6891889 31248666

[4] Hanahan D Weinberg RA. Hallmarks of cancer: the next generation. Cell. (2011) 144:646–74. doi: 10.1016/j.cell.2011.02.013 21376230

[5] Hanahan D Coussens LM. Accessories to the crime: functions of cells recruited to the tumor microenvironment. Cancer Cell. (2012) 21:309–22. doi: 10.1016/j.ccr.2012.02.022 22439926

[6] Quail DF Joyce JA. Microenvironmental regulation of tumor progression and metastasis. Nat Med. (2013) 19:1423–37. doi: 10.1038/nm.3394 PMC3954707 24202395

[7] Croft W Evans RPT Pearce H Elshafie M Griffiths EA Moss P. The single cell transcriptional landscape of esophageal adenocarcinoma and its modulation by neoadjuvant chemotherapy. Mol Cancer. (2022) 21:200. doi: 10.1186/s12943-022-01666-x 36253784 PMC9575245

[8] Hui L Chen Y. Tumor microenvironment: Sanctuary of the devil. Cancer Lett. (2015) 368:7–13. doi: 10.1016/j.canlet.2015.07.039 26276713

[9] Lawson DA Kessenbrock K Davis RT Pervolarakis N Werb Z. Tumour heterogeneity and metastasis at single-cell resolution. Nat Cell Biol. (2018) 20:1349–60. doi: 10.1038/s41556-018-0236-7 PMC6477686 30482943

[10] Hirata E Sahai E. Tumor microenvironment and differential responses to therapy. Cold Spring Harb Perspect Med. (2017) 7:a026781. doi: 10.1101/cshperspect.a026781 28213438 PMC5495051

[11] Faubert B Solmonson A DeBerardinis RJ. Metabolic reprogramming and cancer progression. Science. (2020) 368:eaaw5473. doi: 10.1126/science.aaw5473 32273439 PMC7227780

[12] Viola A Munari F Sánchez-Rodríguez R Scolaro T Castegna A. The metabolic signature of macrophage responses. Front Immunol. (2019) 10:1462. doi: 10.3389/fimmu.2019.01462 31333642 PMC6618143

[13] Zhang J Dong Y Di S Xie S Fan B Gong T. Tumor associated macrophages in esophageal squamous carcinoma: Promising therapeutic implications. BioMed Pharmacother Biomed Pharmacother. (2023) 167:115610. doi: 10.1016/j.biopha.2023.115610 37783153

[14] Liu L-X Heng J-H Deng D-X Zhao H Zheng Z-Y Liao L-D. Sulconazole induces PANoptosis by triggering oxidative stress and inhibiting glycolysis to increase radiosensitivity in esophageal cancer. Mol Cell Proteomics MCP. (2023) 22:100551. doi: 10.1016/j.mcpro.2023.100551 37076047 PMC10205543

[15] Ma G Zhang Z Li P Zhang Z Zeng M Liang Z. Reprogramming of glutamine metabolism and its impact on immune response in the tumor microenvironment. Cell Commun Signal CCS. (2022) 20:114. doi: 10.1186/s12964-022-00909-0 35897036 PMC9327201

[16] Wu H Huang H Zhao Y. Interplay between metabolic reprogramming and post-translational modifications: from glycolysis to lactylation. Front Immunol. (2023) 14:1211221. doi: 10.3389/fimmu.2023.1211221 37457701 PMC10338923

[17] Lim SA Su W Chapman NM Chi H. Lipid metabolism in T cell signaling and function. Nat Chem Biol. (2022) 18:470–81. doi: 10.1038/s41589-022-01017-3 PMC11103273 35484263

[18] Netea-Maier RT Smit JWA Netea MG. Metabolic changes in tumor cells and tumor-associated macrophages: A mutual relationship. Cancer Lett. (2018) 413:102–9. doi: 10.1016/j.canlet.2017.10.037 29111350

[19] Shaul ME Fridlender ZG. Cancer-related circulating and tumor-associated neutrophils - subtypes, sources and function. FEBS J. (2018) 285:4316–42. doi: 10.1111/febs.14524 29851227

[20] Wolf D Sopper S Pircher A Gastl G Wolf AM. Treg(s) in cancer: friends or foe? J Cell Physiol. (2015) 230:2598–605. doi: 10.1002/jcp.25016 25913194

[21] Gabrilovich DI. Myeloid-derived suppressor cells. Cancer Immunol Res. (2017) 5:3–8. doi: 10.1158/2326-6066.CIR-16-0297 28052991 PMC5426480

[22] Hida K Maishi N Torii C Hida Y. Tumor angiogenesis–characteristics of tumor endothelial cells. Int J Clin Oncol. (2016) 21:206–12. doi: 10.1007/s10147-016-0957-1 26879652

[23] Kalluri R Zeisberg M. Fibroblasts in cancer. Nat Rev Cancer. (2006) 6:392–401. doi: 10.1038/nrc1877 16572188

[24] Warburg O. On the origin of cancer cells. Science. (1956) 123:309–14. doi: 10.1126/science.123.3191.309 13298683

[25] Ko P-J Dixon SJ. Protein palmitoylation and cancer. EMBO Rep. (2018) 19:e46666. doi: 10.15252/embr.201846666 30232163 PMC6172454

[26] Li Z Zhang H. Reprogramming of glucose, fatty acid and amino acid metabolism for cancer progression. Cell Mol Life Sci CMLS. (2016) 73:377–92. doi: 10.1007/s00018-015-2070-4 PMC11108301 26499846

[27] Geck RC Toker A. Nonessential amino acid metabolism in breast cancer. Adv Biol Regul. (2016) 62:11–7. doi: 10.1016/j.jbior.2016.01.001 26838061

[28] Salisbury TB Arthur S. The regulation and function of the L-type amino acid transporter 1 (LAT1) in cancer. Int J Mol Sci. (2018) 19:2373. doi: 10.3390/ijms19082373 30103560 PMC6121554

[29] Liu H Zhao J Fu R Zhu C Fan D. The ginsenoside Rk3 exerts anti-esophageal cancer activity *in vitro* and *in vivo* by mediating apoptosis and autophagy through regulation of the PI3K/Akt/mTOR pathway. PloS One. (2019) 14:e0216759. doi: 10.1371/journal.pone.0216759 31091245 PMC6519821

[30] Samson P Lockhart AC. Biologic therapy in esophageal and gastric Malignancies: current therapies and future directions. J Gastrointest Oncol. (2017) 8:418–29. doi: 10.21037/jgo.2016.11.13 PMC5506284 28736629

[31] Tanaka T Nakamura J Noshiro H. Promising immunotherapies for esophageal cancer. Expert Opin Biol Ther. (2017) 17:723–33. doi: 10.1080/14712598.2017.1315404 28366014

[32] Zhang Y Zhang Y Zhang L. Expression of cancer-testis antigens in esophageal cancer and their progress in immunotherapy. J Cancer Res Clin Oncol. (2019) 145:281–91. doi: 10.1007/s00432-019-02840-3 PMC6373256 30656409

[33] Janjigian YY Bendell J Calvo E Kim JW Ascierto PA Sharma P. CheckMate-032 study: efficacy and safety of nivolumab and nivolumab plus ipilimumab in patients with metastatic esophagogastric cancer. J Clin Oncol Off J Am Soc Clin Oncol. (2018) 36:2836–44. doi: 10.1200/JCO.2017.76.6212 PMC6161834 30110194

[34] Kudo T Hamamoto Y Kato K Ura T Kojima T Tsushima T. Nivolumab treatment for oesophageal squamous-cell carcinoma: an open-label, multicentre, phase 2 trial. Lancet Oncol. (2017) 18:631–9. doi: 10.1016/S1470-2045(17)30181-X 28314688

[35] Oya Y Hayakawa Y Koike K. Tumor microenvironment in gastric cancers. Cancer Sci. (2020) 111:2696–707. doi: 10.1111/cas.14521 PMC7419059 32519436

[36] Banchereau J Steinman RM. Dendritic cells and the control of immunity. Nature. (1998) 392:245–52. doi: 10.1038/32588 9521319

[37] Crespo J Sun H Welling TH Tian Z Zou W. T cell anergy, exhaustion, senescence, and stemness in the tumor microenvironment. Curr Opin Immunol. (2013) 25:214–21. doi: 10.1016/j.coi.2012.12.003 PMC3636159 23298609

[38] Ando M Ito M Srirat T Kondo T Yoshimura A. Memory T cell, exhaustion, and tumor immunity. Immunol Med. (2020) 43:1–9. doi: 10.1080/25785826.2019.1698261 31822213

[39] O’Brien KL Finlay DK. Immunometabolism and natural killer cell responses. Nat Rev Immunol. (2019) 19:282–90. doi: 10.1038/s41577-019-0139-2 30808985

[40] Shimada H Takeda A Nabeya Y Okazumi SI Matsubara H Funami Y. Clinical significance of serum vascular endothelial growth factor in esophageal squamous cell carcinoma. Cancer. (2001) 92:663–9. doi: 10.1002/1097-0142(20010801)92:3<663::aid-cncr1368>3.0.co;2-l 11505413

[41] Chen Y Zhu S Liu T Zhang S Lu J Fan W. Epithelial cells activate fibroblasts to promote esophageal cancer development. Cancer Cell. (2023) 41:903–918.e8. doi: 10.1016/j.ccell.2023.03.001 36963399

[42] Chen X Cheng G Zhu L Liu T Yang X Liu R. Alarmin S100A8 imparts chemoresistance of esophageal cancer by reprogramming cancer-associated fibroblasts. Cell Rep Med. (2024) 5:101576. doi: 10.1016/j.xcrm.2024.101576 38776909 PMC11228400

[43] Lavon H Scherz-Shouval R. Insights into the co-evolution of epithelial cells and fibroblasts in the esophageal tumor microenvironment. Cancer Cell. (2023) 41:826–8. doi: 10.1016/j.ccell.2023.03.020 37054715

[44] Qiu L Yue J Ding L Yin Z Zhang K Zhang H. Cancer-associated fibroblasts: An emerging target against esophageal squamous cell carcinoma. Cancer Lett. (2022) 546:215860. doi: 10.1016/j.canlet.2022.215860 35948121

[45] Wang J Zhang G Wang J Wang L Huang X Cheng Y. The role of cancer-associated fibroblasts in esophageal cancer. J Transl Med. (2016) 14:30. doi: 10.1186/s12967-016-0788-x 26822225 PMC4732002

[46] Fujiya T Asanuma K Koike T Okata T Saito M Asano N. Nitric oxide could promote development of Barrett’s esophagus by S-nitrosylation-induced inhibition of Rho-ROCK signaling in esophageal fibroblasts. Am J Physiol Gastrointest Liver Physiol. (2022) 322:G107–16. doi: 10.1152/ajpgi.00124.2021 34786954

[47] Blank S Nienhüser H Dreikhausen L Sisic L Heger U Ott K. Inflammatory cytokines are associated with response and prognosis in patients with esophageal cancer. Oncotarget. (2017) 8:47518–32. doi: 10.18632/oncotarget.17671 PMC5564583 28537901

[48] Cao W Peters JH Nieman D Sharma M Watson T Yu J. Macrophage subtype predicts lymph node metastasis in oesophageal adenocarcinoma and promotes cancer cell invasion *in vitro* . Br J Cancer. (2015) 113:738–46. doi: 10.1038/bjc.2015.292 PMC4559839 26263481

[49] Miyashita T Tajima H Shah FA Oshima M Makino I Nakagawara H. Impact of inflammation-metaplasia-adenocarcinoma sequence and inflammatory microenvironment in esophageal carcinogenesis using surgical rat models. Ann Surg Oncol. (2014) 21:2012–9. doi: 10.1245/s10434-014-3537-5 24526548

[50] Gabitass RF Annels NE Stocken DD Pandha HA Middleton GW. Elevated myeloid-derived suppressor cells in pancreatic, esophageal and gastric cancer are an independent prognostic factor and are associated with significant elevation of the Th2 cytokine interleukin-13. Cancer Immunol Immunother CII. (2011) 60:1419–30. doi: 10.1007/s00262-011-1028-0 PMC3176406 21644036

[51] Karakasheva TA Waldron TJ Eruslanov E Kim S-B Lee J-S O’Brien S. CD38-expressing myeloid-derived suppressor cells promote tumor growth in a murine model of esophageal cancer. Cancer Res. (2015) 75:4074–85. doi: 10.1158/0008-5472.CAN-14-3639 PMC4592477 26294209

[52] Ma Q Xing C Long W Wang HY Liu Q Wang R-F. Impact of microbiota on central nervous system and neurological diseases: the gut-brain axis. J Neuroinflamm. (2019) 16:53. doi: 10.1186/s12974-019-1434-3 PMC6397457 30823925

[53] Yang L Li A Wang Y Zhang Y. Intratumoral microbiota: roles in cancer initiation, development and therapeutic efficacy. Signal Transduct Target Ther. (2023) 8:35. doi: 10.1038/s41392-022-01304-4 36646684 PMC9842669

[54] Matsuda H Iwahori K Takeoka T Kato R Urakawa S Saito T. Helicobacter pylori infection affects the tumor immune microenvironment of esophageal cancer patients. Anticancer Res. (2024) 44:3799–805. doi: 10.21873/anticanres.17205 39197894

[55] Wu H Leng X Liu Q Mao T Jiang T Liu Y. Intratumoral microbiota composition regulates chemoimmunotherapy response in esophageal squamous cell carcinoma. Cancer Res. (2023) 83:3131–44. doi: 10.1158/0008-5472.CAN-22-2593 37433041

[56] Vaupel P Schmidberger H Mayer A. The Warburg effect: essential part of metabolic reprogramming and central contributor to cancer progression. Int J Radiat Biol. (2019) 95:912–9. doi: 10.1080/09553002.2019.1589653 30822194

[57] Hochwald JS Zhang J. Glucose oncometabolism of esophageal cancer. Anticancer Agents Med Chem. (2017) 17:385–94. doi: 10.2174/1871520616666160627092716 27357541

[58] Angelin A Gil-de-Gómez L Dahiya S Jiao J Guo L Levine MH. Foxp3 reprograms T cell metabolism to function in low-glucose, high-lactate environments. Cell Metab. (2017) 25:1282–1293.e7. doi: 10.1016/j.cmet.2016.12.018 28416194 PMC5462872

[59] Fang H-Y Hughes R Murdoch C Coffelt SB Biswas SK Harris AL. Hypoxia-inducible factors 1 and 2 are important transcriptional effectors in primary macrophages experiencing hypoxia. Blood. (2009) 114:844–59. doi: 10.1182/blood-2008-12-195941 PMC2882173 19454749

[60] Wang T Liu H Lian G Zhang S-Y Wang X Jiang C. HIF1α-induced glycolysis metabolism is essential to the activation of inflammatory macrophages. Mediators Inflammation. (2017) 2017:9029327. doi: 10.1155/2017/9029327 PMC5745720 29386753

[61] Bader JE Voss K Rathmell JC. Targeting metabolism to improve the tumor microenvironment for cancer immunotherapy. Mol Cell. (2020) 78:1019–33. doi: 10.1016/j.molcel.2020.05.034 PMC7339967 32559423

[62] Lu Z-N Song J Sun T-H Sun G. UBE2C affects breast cancer proliferation through the AKT/mTOR signaling pathway. Chin Med J (Engl). (2021) 134:2465–74. doi: 10.1097/CM9.0000000000001708 PMC8654430 34620747

[63] Buckley AM Lynam-Lennon N O’Neill H O’Sullivan J. Targeting hallmarks of cancer to enhance radiosensitivity in gastrointestinal cancers. Nat Rev Gastroenterol Hepatol. (2020) 17:298–313. doi: 10.1038/s41575-019-0247-2 32005946

[64] Luo W Semenza GL. Emerging roles of PKM2 in cell metabolism and cancer progression. Trends Endocrinol Metab TEM. (2012) 23:560–6. doi: 10.1016/j.tem.2012.06.010 PMC3466350 22824010

[65] Ma R Liu Q Zheng S Liu T Tan D Lu X. PKM2-regulated STAT3 promotes esophageal squamous cell carcinoma progression via TGF-β1-induced EMT. J Cell Biochem. (2019) 120:11539–50. doi: 10.1002/jcb.28434 30756445

[66] Feng J Wu L Ji J Chen K Yu Q Zhang J. PKM2 is the target of proanthocyanidin B2 during the inhibition of hepatocellular carcinoma. J Exp Clin Cancer Res CR. (2019) 38:204. doi: 10.1186/s13046-019-1194-z 31101057 PMC6525465

[67] Kim J Tchernyshyov I Semenza GL Dang CV. HIF-1-mediated expression of pyruvate dehydrogenase kinase: a metabolic switch required for cellular adaptation to hypoxia. Cell Metab. (2006) 3:177–85. doi: 10.1016/j.cmet.2006.02.002 16517405

[68] Palsson-McDermott EM Curtis AM Goel G Lauterbach MAR Sheedy FJ Gleeson LE. Pyruvate kinase M2 regulates Hif-1α activity and IL-1β induction and is a critical determinant of the warburg effect in LPS-activated macrophages. Cell Metab. (2015) 21:65–80. doi: 10.1016/j.cmet.2014.12.005 25565206 PMC5198835

[69] Mazzone M Menga A Castegna A. Metabolism and TAM functions-it takes two to tango. FEBS J. (2018) 285:700–16. doi: 10.1111/febs.14295 29055087

[70] Eisinger S Sarhan D Boura VF Ibarlucea-Benitez I Tyystjärvi S Oliynyk G. Targeting a scavenger receptor on tumor-associated macrophages activates tumor cell killing by natural killer cells. Proc Natl Acad Sci U.S.A. (2020) 117:32005–16. doi: 10.1073/pnas.2015343117 PMC7750482 33229588

[71] Colegio OR Chu N-Q Szabo AL Chu T Rhebergen AM Jairam V. Functional polarization of tumour-associated macrophages by tumour-derived lactic acid. Nature. (2014) 513:559–63. doi: 10.1038/nature13490 PMC4301845 25043024

[72] Vitale I Manic G Coussens LM Kroemer G Galluzzi L. Macrophages and metabolism in the tumor microenvironment. Cell Metab. (2019) 30:36–50. doi: 10.1016/j.cmet.2019.06.001 31269428

[73] Chen P Huang Y Bong R Ding Y Song N Wang X. Tumor-associated macrophages promote angiogenesis and melanoma growth via adrenomedullin in a paracrine and autocrine manner. Clin Cancer Res Off J Am Assoc Cancer Res. (2011) 17:7230–9. doi: 10.1158/1078-0432.CCR-11-1354 21994414

[74] Hughes R Qian B-Z Rowan C Muthana M Keklikoglou I Olson OC. Perivascular M2 macrophages stimulate tumor relapse after chemotherapy. Cancer Res. (2015) 75:3479–91. doi: 10.1158/0008-5472.CAN-14-3587 PMC5024531 26269531

[75] Voss K Larsen SE Snow AL. Metabolic reprogramming and apoptosis sensitivity: Defining the contours of a T cell response. Cancer Lett. (2017) 408:190–6. doi: 10.1016/j.canlet.2017.08.033 PMC5628155 28866092

[76] Mojsilovic SS Mojsilovic S Villar VH Santibanez JF. The metabolic features of tumor-associated macrophages: opportunities for immunotherapy? Anal Cell Pathol Amst. (2021) 2021:5523055. doi: 10.1155/2021/5523055 34476174 PMC8407977

[77] Li W Tanikawa T Kryczek I Xia H Li G Wu K. Aerobic glycolysis controls myeloid-derived suppressor cells and tumor immunity via a specific CEBPB isoform in triple-negative breast cancer. Cell Metab. (2018) 28:87–103.e6. doi: 10.1016/j.cmet.2018.04.022 29805099 PMC6238219

[78] Macintyre AN Gerriets VA Nichols AG Michalek RD Rudolph MC Deoliveira D. The glucose transporter Glut1 is selectively essential for CD4 T cell activation and effector function. Cell Metab. (2014) 20:61–72. doi: 10.1016/j.cmet.2014.05.004 24930970 PMC4079750

[79] Zhang H Xie C Yue J Jiang Z Zhou R Xie R. Cancer-associated fibroblasts mediated chemoresistance by a FOXO1/TGFβ1 signaling loop in esophageal squamous cell carcinoma. Mol Carcinog. (2017) 56:1150–63. doi: 10.1002/mc.22581 27769097

[80] Blum AE Venkitachalam S Ravillah D Chelluboyina AK Kieber-Emmons AM Ravi L. Systems biology analyses show hyperactivation of transforming growth factor-β and JNK signaling pathways in esophageal cancer. Gastroenterology. (2019) 156:1761–74. doi: 10.1053/j.gastro.2019.01.263 PMC6701681 30768984

[81] Chen J Gingold JA Su X. Immunomodulatory TGF-β Signaling in hepatocellular carcinoma. Trends Mol Med. (2019) 25:1010–23. doi: 10.1016/j.molmed.2019.06.007 31353124

[82] Vinocha A Grover RK Deepak R. Clinical significance of interleukin-6 in diagnosis of lung, oral, esophageal, and gall bladder carcinomas. J Cancer Res Ther. (2018) 14:S758–60. doi: 10.4103/0973-1482.183217 30249899

[83] Ebbing EA van der Zalm AP Steins A Creemers A Hermsen S Rentenaar R. Stromal-derived interleukin 6 drives epithelial-to-mesenchymal transition and therapy resistance in esophageal adenocarcinoma. Proc Natl Acad Sci U.S.A. (2019) 116:2237–42. doi: 10.1073/pnas.1820459116 PMC6369811 30670657

[84] Sivanand S Vander Heiden MG. Emerging roles for branched-chain amino acid metabolism in cancer. Cancer Cell. (2020) 37:147–56. doi: 10.1016/j.ccell.2019.12.011 PMC7082774 32049045

[85] Chen C-L Hsu S-C Ann DK Yen Y Kung H-J. Arginine signaling and cancer metabolism. Cancers. (2021) 13:3541. doi: 10.3390/cancers13143541 34298755 PMC8306961

[86] Keshet R Erez A. Arginine and the metabolic regulation of nitric oxide synthesis in cancer. Dis Model Mech. (2018) 11:dmm033332. doi: 10.1242/dmm.033332 30082427 PMC6124554

[87] Mieulet V Yan L Choisy C Sully K Procter J Kouroumalis A. TPL-2-mediated activation of MAPK downstream of TLR4 signaling is coupled to arginine availability. Sci Signal. (2010) 3:ra61. doi: 10.1126/scisignal.2000934 20716763

[88] Sun W Kou H Fang Y Xu F Xu Z Wang X. FOXO3a-regulated arginine metabolic plasticity adaptively promotes esophageal cancer proliferation and metastasis. Oncogene. (2024) 43:216–23. doi: 10.1038/s41388-023-02906-0 38049565

[89] Altman BJ Stine ZE Dang CV. From Krebs to clinic: glutamine metabolism to cancer therapy. Nat Rev Cancer. (2016) 16:619–34. doi: 10.1038/nrc.2016.71 PMC5484415 27492215

[90] Cluntun AA Lukey MJ Cerione RA Locasale JW. Glutamine metabolism in cancer: understanding the heterogeneity. Trends Cancer. (2017) 3:169–80. doi: 10.1016/j.trecan.2017.01.005 PMC5383348 28393116

[91] Yuneva M Zamboni N Oefner P Sachidanandam R Lazebnik Y. Deficiency in glutamine but not glucose induces MYC-dependent apoptosis in human cells. J Cell Biol. (2007) 178:93–105. doi: 10.1083/jcb.200703099 17606868 PMC2064426

[92] Hensley CT Wasti AT DeBerardinis RJ. Glutamine and cancer: cell biology, physiology, and clinical opportunities. J Clin Invest. (2013) 123:3678–84. doi: 10.1172/JCI69600 PMC3754270 23999442

[93] Yang L Venneti S Nagrath D. Glutaminolysis: A hallmark of cancer metabolism. Annu Rev BioMed Eng. (2017) 19:163–94. doi: 10.1146/annurev-bioeng-071516-044546 28301735

[94] Daye D Wellen KE. Metabolic reprogramming in cancer: unraveling the role of glutamine in tumorigenesis. Semin Cell Dev Biol. (2012) 23:362–9. doi: 10.1016/j.semcdb.2012.02.002 22349059

[95] Wise DR DeBerardinis RJ Mancuso A Sayed N Zhang X-Y Pfeiffer HK. Myc regulates a transcriptional program that stimulates mitochondrial glutaminolysis and leads to glutamine addiction. Proc Natl Acad Sci U.S.A. (2008) 105:18782–7. doi: 10.1073/pnas.0810199105 PMC2596212 19033189

[96] Gao P Tchernyshyov I Chang T-C Lee Y-S Kita K Ochi T. c-Myc suppression of miR-23a/b enhances mitochondrial glutaminase expression and glutamine metabolism. Nature. (2009) 458:762–5. doi: 10.1038/nature07823 PMC2729443 19219026

[97] Hu W Zhang C Wu R Sun Y Levine A Feng Z. Glutaminase 2, a novel p53 target gene regulating energy metabolism and antioxidant function. Proc Natl Acad Sci U.S.A. (2010) 107:7455–60. doi: 10.1073/pnas.1001006107 PMC2867677 20378837

[98] Takeuchi Y Nakayama Y Fukusaki E Irino Y. Glutamate production from ammonia via glutamate dehydrogenase 2 activity supports cancer cell proliferation under glutamine depletion. Biochem Biophys Res Commun. (2018) 495:761–7. doi: 10.1016/j.bbrc.2017.11.088 29146184

[99] Borodovsky A Seltzer MJ Riggins GJ. Altered cancer cell metabolism in gliomas with mutant IDH1 or IDH2. Curr Opin Oncol. (2012) 24:83–9. doi: 10.1097/CCO.0b013e32834d816a PMC4612588 22080945

[100] Liu J Zhang C Lin M Zhu W Liang Y Hong X. Glutaminase 2 negatively regulates the PI3K/AKT signaling and shows tumor suppression activity in human hepatocellular carcinoma. Oncotarget. (2014) 5:2635–47. doi: 10.18632/oncotarget.1862 PMC4058033 24797434

[101] Zhao L Huang Y Zheng J. STAT1 regulates human glutaminase 1 promoter activity through multiple binding sites in HIV-1 infected macrophages. PloS One. (2013) 8:e76581. doi: 10.1371/journal.pone.0076581 24086752 PMC3782442

[102] Zhao L Huang Y Tian C Taylor L Curthoys N Wang Y. Interferon-α regulates glutaminase 1 promoter through STAT1 phosphorylation: relevance to HIV-1 associated neurocognitive disorders. PloS One. (2012) 7:e32995. doi: 10.1371/journal.pone.0032995 22479354 PMC3316554

[103] Thangavelu K Pan CQ Karlberg T Balaji G Uttamchandani M Suresh V. Structural basis for the allosteric inhibitory mechanism of human kidney-type glutaminase (KGA) and its regulation by Raf-Mek-Erk signaling in cancer cell metabolism. Proc Natl Acad Sci U.S.A. (2012) 109:7705–10. doi: 10.1073/pnas.1116573109 PMC3356676 22538822

[104] Son J Lyssiotis CA Ying H Wang X Hua S Ligorio M. Glutamine supports pancreatic cancer growth through a KRAS-regulated metabolic pathway. Nature. (2013) 496:101–5. doi: 10.1038/nature12040 PMC3656466 23535601

[105] Zhu Y Lin G Dai Z Zhou T Li T Yuan T. L-Glutamine deprivation induces autophagy and alters the mTOR and MAPK signaling pathways in porcine intestinal epithelial cells. Amino Acids. (2015) 47:2185–97. doi: 10.1007/s00726-014-1785-0 24997162

[106] Ma EH Dahabieh MS DeCamp LM Kaymak I Kitchen-Goosen SM Oswald BM. 13C metabolite tracing reveals glutamine and acetate as critical *in vivo* fuels for CD8 T cells. Sci Adv. (2024) 10:eadj1431. doi: 10.1126/sciadv.adj1431 38809979 PMC11135420

[107] Carr EL Kelman A Wu GS Gopaul R Senkevitch E Aghvanyan A. Glutamine uptake and metabolism are coordinately regulated by ERK/MAPK during T lymphocyte activation. J Immunol Baltim Md 1950. (2010) 185:1037–44. doi: 10.4049/jimmunol.0903586 PMC2897897 20554958

[108] Pallett LJ Dimeloe S Sinclair LV Byrne AJ Schurich A. A glutamine “tug-of-war”: targets to manipulate glutamine metabolism for cancer immunotherapy. Immunother Adv. (2021) 1:ltab010. doi: 10.1093/immadv/ltab010 34541580 PMC8444990

[109] Edwards DN Ngwa VM Raybuck AL Wang S Hwang Y Kim LC. Selective glutamine metabolism inhibition in tumor cells improves antitumor T lymphocyte activity in triple-negative breast cancer. J Clin Invest. (2021) 131:e140100, 140100. doi: 10.1172/JCI140100 33320840 PMC7880417

[110] Gross MI Demo SD Dennison JB Chen L Chernov-Rogan T Goyal B. Antitumor activity of the glutaminase inhibitor CB-839 in triple-negative breast cancer. Mol Cancer Ther. (2014) 13:890–901. doi: 10.1158/1535-7163.MCT-13-0870 24523301

[111] Johnson MO Wolf MM Madden MZ Andrejeva G Sugiura A Contreras DC. Distinct regulation of th17 and th1 cell differentiation by glutaminase-dependent metabolism. Cell. (2018) 175:1780–1795.e19. doi: 10.1016/j.cell.2018.10.001 30392958 PMC6361668

[112] Schulte ML Fu A Zhao P Li J Geng L Smith ST. Pharmacological blockade of ASCT2-dependent glutamine transport leads to antitumor efficacy in preclinical models. Nat Med. (2018) 24:194–202. doi: 10.1038/nm.4464 29334372 PMC5803339

[113] Cheng C Geng F Cheng X Guo D. Lipid metabolism reprogramming and its potential targets in cancer. Cancer Commun Lond Engl. (2018) 38:27. doi: 10.1186/s40880-018-0301-4 PMC5993136 29784041

[114] Zaidi N Swinnen JV Smans K. ATP-citrate lyase: a key player in cancer metabolism. Cancer Res. (2012) 72:3709–14. doi: 10.1158/0008-5472.CAN-11-4112 22787121

[115] Hanai J Doro N Sasaki AT Kobayashi S Cantley LC Seth P. Inhibition of lung cancer growth: ATP citrate lyase knockdown and statin treatment leads to dual blockade of mitogen-activated protein kinase (MAPK) and phosphatidylinositol-3-kinase (PI3K)/AKT pathways. J Cell Physiol. (2012) 227:1709–20. doi: 10.1002/jcp.22895 PMC3407542 21688263

[116] Granchi C. ATP citrate lyase (ACLY) inhibitors: An anti-cancer strategy at the crossroads of glucose and lipid metabolism. Eur J Med Chem. (2018) 157:1276–91. doi: 10.1016/j.ejmech.2018.09.001 30195238

[117] Hopperton KE Duncan RE Bazinet RP Archer MC. Fatty acid synthase plays a role in cancer metabolism beyond providing fatty acids for phospholipid synthesis or sustaining elevations in glycolytic activity. Exp Cell Res. (2014) 320:302–10. doi: 10.1016/j.yexcr.2013.10.016 24200503

[118] Menendez JA Lupu R. Fatty acid synthase and the lipogenic phenotype in cancer pathogenesis. Nat Rev Cancer. (2007) 7:763–77. doi: 10.1038/nrc2222 17882277

[119] Bensaad K Favaro E Lewis CA Peck B Lord S Collins JM. Fatty acid uptake and lipid storage induced by HIF-1α contribute to cell growth and survival after hypoxia-reoxygenation. Cell Rep. (2014) 9:349–65. doi: 10.1016/j.celrep.2014.08.056 25263561

[120] Wang H Franco F Tsui Y-C Xie X Trefny MP Zappasodi R. CD36-mediated metabolic adaptation supports regulatory T cell survival and function in tumors. Nat Immunol. (2020) 21:298–308. doi: 10.1038/s41590-019-0589-5 32066953 PMC7043937

[121] Sun Y He W Luo M Zhou Y Chang G Ren W. SREBP1 regulates tumorigenesis and prognosis of pancreatic cancer through targeting lipid metabolism. Tumour Biol J Int Soc Oncodevelopmental Biol Med. (2015) 36:4133–41. doi: 10.1007/s13277-015-3047-5 25589463

[122] Zhang G-C Yu X-N Guo H-Y Sun J-L Liu Z-Y Zhu J-M. PRP19 enhances esophageal squamous cell carcinoma progression by reprogramming SREBF1-dependent fatty acid metabolism. Cancer Res. (2023) 83:521–37. doi: 10.1158/0008-5472.CAN-22-2156 36723974

[123] Ma Y Temkin SM Hawkridge AM Guo C Wang W Wang X-Y. Fatty acid oxidation: An emerging facet of metabolic transformation in cancer. Cancer Lett. (2018) 435:92–100. doi: 10.1016/j.canlet.2018.08.006 30102953 PMC6240910

[124] Tian T Lu Y Lin J Chen M Qiu H Zhu W. CPT1A promotes anoikis resistance in esophageal squamous cell carcinoma via redox homeostasis. Redox Biol. (2022) 58:102544. doi: 10.1016/j.redox.2022.102544 36427397 PMC9692043

[125] Wang PP Song X Zhao XK Wei MX Gao SG Zhou FY. Serum metabolomic profiling reveals biomarkers for early detection and prognosis of esophageal squamous cell carcinoma. Front Oncol. (2022) 12:790933. doi: 10.3389/fonc.2022.790933 35155234 PMC8832491

[126] Raud B Roy DG Divakaruni AS Tarasenko TN Franke R Ma EH. Etomoxir actions on regulatory and memory T cells are independent of cpt1a-mediated fatty acid oxidation. Cell Metab. (2018) 28:504–515.e7. doi: 10.1016/j.cmet.2018.06.002 30043753 PMC6747686

[127] Su P Wang Q Bi E Ma X Liu L Yang M. Enhanced lipid accumulation and metabolism are required for the differentiation and activation of tumor-associated macrophages. Cancer Res. (2020) 80:1438–50. doi: 10.1158/0008-5472.CAN-19-2994 PMC7127942 32015091

[128] Tardito S Oudin A Ahmed SU Fack F Keunen O Zheng L. Glutamine synthetase activity fuels nucleotide biosynthesis and supports growth of glutamine-restricted glioblastoma. Nat Cell Biol. (2015) 17:1556–68. doi: 10.1038/ncb3272 PMC4663685 26595383

[129] Zhang D Rubio Rodríguez-Kirby LA Lin Y Song M Wang L Wang L. Spatial dynamics of mammalian brain development and neuroinflammation by multimodal tri-omics mapping. BioRxiv Prepr Serv Biol. (2024), 2024.07.28.605493. doi: 10.1101/2024.07.28.605493

[130] Baysoy A Tian X Zhang F Renauer P Bai Z Shi H. Spatially Resolved *in vivo* CRISPR Screen Sequencing via Perturb-DBiT. BioRxiv Prepr Serv Biol. (2024), 2024.11.18.624106. doi: 10.1101/2024.11.18.624106

[131] Liu Y DiStasio M Su G Asashima H Enninful A Qin X. High-plex protein and whole transcriptome co-mapping at cellular resolution with spatial CITE-seq. Nat Biotechnol. (2023) 41:1405–9. doi: 10.1038/s41587-023-01676-0 PMC10567548 36823353

[132] Kelly RJ. Immunotherapy for esophageal and gastric cancer. Am Soc Clin Oncol Educ Book Am Soc Clin Oncol Annu Meet. (2017) 37:292–300. doi: 10.1200/EDBK_175231 28561677

